# Robotic-assisted treatment of flank hernias with progressive intracorporeal fascial traction (PIFT): A novel technique for secure fascial adaptation

**DOI:** 10.1007/s10029-025-03527-0

**Published:** 2025-11-19

**Authors:** Anna Hannebauer, Ahmed Al-Mawsheki, Maximilian Bockhorn, Fadl Alfarawan

**Affiliations:** 1 Fakultät für Gesundheitswissenschaften, Carl von Ossietzky Universität Oldenburg, Ammerländer Heerstraße 114-118, Oldenburg, 26129 Germany; 2 Department for General – and Visceral Surgery, University Hospital Oldenburg, Klinikum Oldenburg AöR, Rahel-Strauss-Straße 10, 26133 Oldenburg, Germany

**Keywords:** Incisional flank hernia, Robotic surgery, Intracorporeal fascial traction, Sliding knot technique, Preperitoneal mesh repair

## Abstract

**Background:**

Incisional flank hernias pose a particular challenge due to scarred, retracted fascial edges and complex regional anatomy. This study evaluates a robotic-assisted technique employing progressive intermittent intracorporeal fascial traction (Vicryl 0 sliding-knot) combined with barbed-suture augmentation (STRATAFIX™ Symmetric PDS 0) in large-volume defects.

**Methods:**

Between June 2023 and January 2025, 13 patients with symptomatic incisional flank hernias underwent repair with the da Vinci X^®^ system. A macroporous polypropylene mesh was implanted. Patients were followed up at 1-, 3-, and 6-months including ultrasound, VAS pain scoring, and recurrence screening.

**Results:**

Median total operative time was 167 min (IQR 117–193.5), console time 141 min (IQR 104.5–176.5). The median defect size was 138.75 cm² [IQR 55.5–249]. Meshes with a median size of 433 cm² [315-572.5] and a mesh-to-defect ratio of > 3:1 were implanted. Pain scores remained stable at median VAS 2/10 on postoperative days 1–3. One patient developed seroma (Clavien-Dindo I); no Clavien-Dindo ≥ II complications occurred. No hernia recurrences were observed till the six months follow-up. Median length of stay was 2 days (IQR 2–3).

**Conclusion:**

Progressive intracorporeal fascial traction with sliding-knot technique and barbed-suture reinforcement allows safe, tension-reduced repair of large incisional flank hernias, resulting in low pain levels, brief hospitalization, and no early recurrences. Longer-term, comparative studies are warranted.

**Supplementary Information:**

The online version contains supplementary material available at 10.1007/s10029-025-03527-0.

## Introduction

Flank hernias are uncommon but clinically significant. They often follow thoracoabdominal or lumbar surgeries and are complicated by scar retraction of fascial edges, proximity to bony landmarks, and neurovascular bundles. Open repair permits direct fascial closure but carries high wound-related morbidity, while laparoscopic intraperitoneal onlay mesh procedures (IPOM) reduce wound complications yet often fail to achieve durable fascial adaptation and entail risks of mesh bulging and seroma formation [1, 2]. Robot-assisted approaches have gained popularity for ventral and incisional hernias, offering 3D visualization and EndoWrist instrumentation that facilitate precise dissection and suturing [3–5]. Several systematic reviews and meta-analyses have demonstrated the safety and efficacy of robotic ventral hernia repair, with reduced length of stay and comparable complication rates versus open and laparoscopic techniques [6]. However, data specific to flank hernias remains sparse. Di Giuseppe et al. first reported a robotic series of seven flank hernias in 2020, confirming feasibility but without targeted fascial-adaptation strategies [7, 8]. Recent innovations include intraoperative fascial traction devices that gradually approximate the fascial edges, avoiding extensive component separations [9]. Bloemendaal et al. described early experiences with minimally invasive fascial distancing in lateral abdominal wall hernias and Kudsi et al. highlighted the importance of standardized outcome reporting for robotic repairs [4, 10]. Barbed sutures have further enhanced abdominal wall reconstructions by distributing tension evenly along the suture line [11, 12]. Building on these principles, we developed a progressive intermittent intracorporeal fascial traction technique using Vicryl 0 sliding-knot sutures, followed by barbed-suture reinforcement (STRATAFIX™ Symmetric PDS 0). Here, we present our initial 13-patient experience with this tension-reducing approach in incisional flank hernias. To the best of our knowledge, this is the first series of robotic-assisted flank hernia repair with progressive intermittent intracorporeal fascial traction combined with barbed-suture reinforcement.

## Materials and methods

### Patient cohort

We retrospectively identified 13 consecutive patients (8 male, 5 female; median age = 67 years [IQR 62–76]; median BMI = 31 kg/m² [IQR 25–34]; ASA II: *n* = 5; ASA III: *n* = 8) who underwent robotic repair of symptomatic incisional flank hernias between June 2023 and January 2025. For further patient demographics see Table 1. Inclusion criteria were incisional flank hernias ≥ 50 cm² post prior flank surgery with clinical symptoms. Exclusion criteria included active infection, prohibitive comorbidities (ASA IV) and relaxation of the abdominal wall. All patients underwent standardized preoperative work-up including clinical examination, ultrasound and computed tomography. If there were any contraindications for Computed Tomography, Magnet Resonance Imaging was conducted. Pre- as well as postoperative work up including ultrasound was conducted by a specialized hernia team. All operations were performed by a senior surgeon with experience in more than 100 robotic hernia procedures at our university hospital where more than 600 hernia procedures overall and more than 200 laparoscopic ventral hernia procedures are performed per year.

**Table 1:** Patient demographics

	Median [IQR]	*n* (%)
Sex		
Male		8 (61.5%)
Female		5 (38.5%)
Age	67 [62–76]	
ASA Classification		
ASA II		5 (38.5%)
ASA III		8 (61.5%)
BMI (kg/m²)	31 [25–34]	
Hernia Type		
Primary		0
Incisional		13 (100%)
Recurrent		0
EHS width		
W1		0
W2		1 (7.7%)
W3		11 (84.6%)
Missing		1 (7.7%)
Defect size (cm²) Missing *n* = 1	138.75 [55.5–249]	

*ASA = American Society of Anesthesiologists Classification, EHS = European Hernia Society*

### Surgical technique

All operations were performed with the da Vinci X^®^ system under general anesthesia with patients in lateral decubitus position and Trendelenburg position (Fig. 2a).

**Fig. 1:**
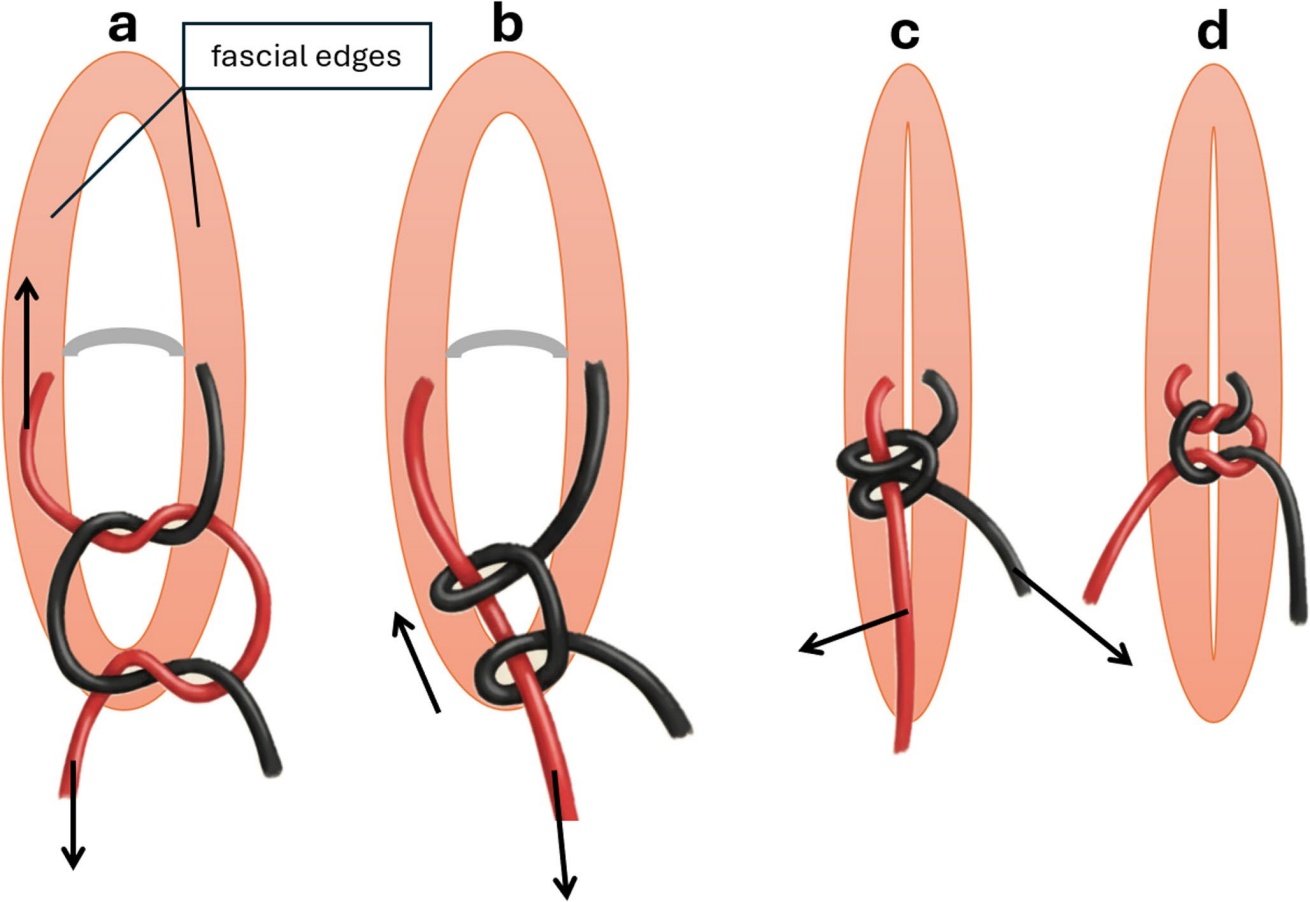
Technique of an intracorporeal slipknot

**Fig. 2:**
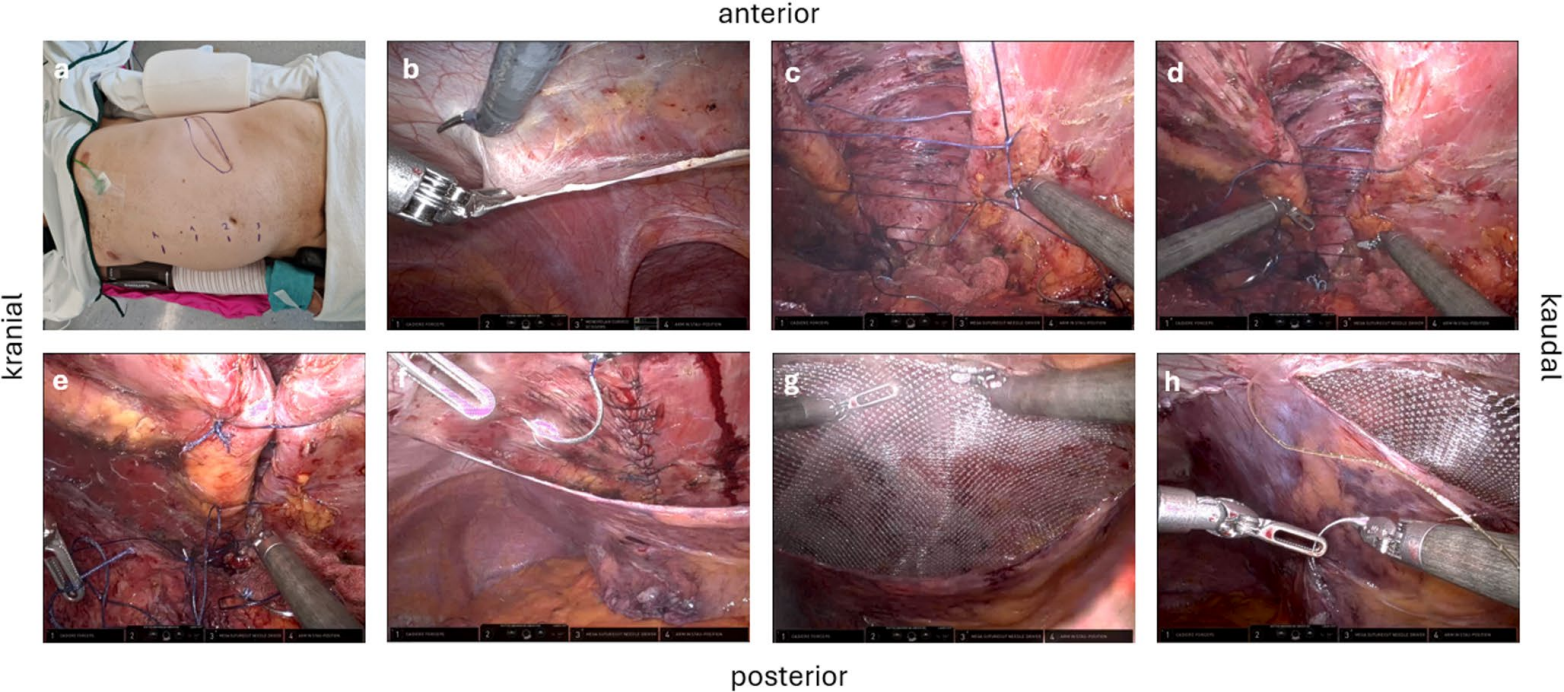
Intraoperative steps of robotic assisting Flank hernia reconstruction

Key steps:

1. Pneumoperitoneum: Established to 10–12 mm Hg via Visio-Port or open technique.
2. Port Placement: Three 8 mm robotic ports ipsilateral to the hernia; one 11 mm assistant port, ≥ 6 cm apart.
3. Preperitoneal Dissection: Creation of a pocket around the defect; excision of hernia sac and adhesions (Fig. 2b).
4. Suture Placement with Initial Sliding Knot Configuration: For the fascial traction sutures, a strong, braided, absorbable suture material (e.g., polyglactin 910, size 2) is used. To enable controlled, stepwise tension adjustment, a sliding knot technique is employed. The sutures are placed as interrupted single stitches through the fascial edges, spanning the hernia defect transversely at intervals of approximately 4 cm. At each suture point, a modified square knot is tied intracorporeally and then deliberately converted into a sliding configuration (“tilted”) by pulling on one suture end. The longer limb of the suture functions as the tensioning limb, while the shorter limb is used later for securing the knot permanently. Initially, the knots are left loose, maintaining only minimal tension across the defect. This ensures even distribution of the sutures across the fascial gap before applying any significant traction, thereby avoiding localized “cheese-wiring” through the tissue. The entire hernia defect is bridged in this manner prior to the progressive closure phase (Fig. 1a and 1b) (Fig. 2c and 2d).
5. Progressive Fascial Closure with Sequential Sliding-Knot Tensioning: Following suture placement, the sliding knots are progressively tightened in sequential fashion at time intervals of approximately 15 min. Each suture is gradually tensioned to approximate the fascial edges under controlled conditions. If signs of excessive tension or tissue cut-through are observed, traction on that particular suture is immediately paused, and tensioning proceeds with the next one. When required, intra-abdominal pressure can be temporarily reduced to 6–8 mm Hg to decrease tension on the Myofascial edges and facilitate approximation. After completion of fascial closure, pneumoperitoneum is re-established to 10–12 mm Hg for inspection and mesh placement. This staged tightening protocol exploits the viscoelastic properties of the abdominal wall fascia, particularly creep (gradual tissue elongation under sustained load) and stress relaxation (reduction of tension over time at a fixed length). As the fascia adapts under load, the edges can be brought together with progressively less force, reducing the risk of tearing or ischemia. In parallel, a continuous, barbed PDS 0 suture (Stratafix^®^) is placed along both the ventral and dorsal fascial edges, spanning the entire defect. As the sliding-knot sutures are sequentially tightened, the continuous sutures are alternately advanced to maintain symmetric closure. Once complete fascial approximation is achieved under significantly reduced peak tension, the sliding knots are secured by adding additional throws, and the barbed sutures are tied to finalize the closure (Fig. 1c and 1d) (Fig. 2e and 2f). This technique enables primary closure of large fascial defects that would otherwise require component separation, by distributing traction forces both spatially (across multiple sutures) and temporally (over successive tightening intervals). Particularly in lateral abdominal wall hernias, where defects are often transverse and the muscle fibers run obliquely or horizontally, aligning the closure vector cranio-caudally allows exploitation of the natural compliance of the abdominal wall. Gradual, controlled traction leads to progressive elongation of the fascia, analogous to external traction systems or progressive pneumoperitoneum, but achieved here through a precise, intracorporeal suture-based mechanism.

(a) Suture placement and knot formation: *A strong, absorbable suture (e.g.,polyglactin) is placed on both sides of the fascial edges. The two suture ends (red and black) are crossed to form the base of the knot. The red end is looped a second time around the black end, analogous to a classic square knot. The knot is shaped by pulling both ends in opposite directions but is not tightened yet. *(b)* Creation of the sliding configuration: By pulling on the red end, the square knot is converted into a sliding configuration. The knot remains loose on the fascia and can be moved freely along the suture. *(c)* Knot advancement and fascial closure: By pulling on the red end, the knot is advanced downward, gradually approximating the fascial edges. The knot can be repeatedly tightened to achieve a controlled, tension-reduced approximation.*(d)* Knot blocking: Finally, both suture ends are pulled simultaneously to block and secure the slipknot.*

6. Mesh Implantation: Heavyweight macroporous polypropylene mesh (median 433 cm² [315–572.5]; mesh-to-defect ratio 3.1:1) was placed preperitoneally and secured with 3 − 0 Vicryl sutures (Fig. 2g).
7. Peritoneal Closure: Completed with STRATAFIX™ 3 − 0 over the mesh (Fig. 2h).

A detailed procedural demonstration is provided in the supplementary material.

*(a) Patient positioning in a semi-lateral position on a vacuum mattress and trocar placement (three 8-mm trocars and one 11-mm assistant trocar). (b) Peritoneal incision approximately 6 cm medial to the hernia. (c - e) Abdominal wall reconstruction using interrupted resorbable sutures (size 2) with sliding-lock knots*,* tightened at controlled intervals. (f) Continuous abdominal wall closure with PDS Stratafix sutures. (g) Placement of a 20 × 25 cm polypropylene mesh. (h) Continuous peritoneal closure with 3 − 0 Monocryl Stratafix sutures.*

## Postoperative management and Follow-up

Patients received standardized multimodal analgesia according to the WHO scheme and early mobilization. At a clinical and sonographic follow-up at 1-, 3- and 6-months seroma detection, and hernia recurrence screening and pain according to VAS were assessed (see Table 2) by the same hernia team that identified the patients beforehand and indicated surgery.

## Results

**Table 2:** Postoperative outcomes

	Median [IQR]	*n* (%)
Hospital Stay (days)	2.5 [2–3]	
Pain		
Postoperative Day 1	2.0 [1–3]	
Postoperative Day 2	2.29 [0–3]	
Postoperative Day 3	1.75 [1–3]	
Surgical Site Occurence		3 (23.1%)
Seroma		3 (23.1%)
Surgical Site Infection		0
Postoperative Ileus		0
Clavien Dindo Classification ≥ II		0

*IQR = interquartile range*

All 13 procedures were completed robotically with no conversions. Surgeries were completed in a median time of 167 min (IQR 117–193.5) and console time of 141 min (IQR 104.5–176.5). The median mesh size was 433 cm² [315–572.5] with a mesh-to-defect ratio > 3:1. No serious complications or early recurrences were observed. The median length of hospital stay was 2 days. Pain was at a median of 1.75–2.29 on VAS scale through postoperative days 1 to 3 (see Table 2).

## Discussion

Our experience demonstrates that progressive intracorporeal fascial traction (PIFT) enables safe and reproducible primary closure of large incisional flank hernias without the need for open component separation [9, 13, 14]. Stepwise tightening of intracorporeal sliding knots facilitates controlled approximation of retracted fascial edges under reduced tension, minimizing the risk of fascial tear-through, suture line failure, or seroma formation. Reinforcement with barbed PDS sutures (STRATAFIX™) distributes tension evenly across the closure line, enhancing mechanical stability and reducing focal stress.

These findings are in line with recent literature on robotic hernia repair, which highlights shorter hospital stays and complication rates comparable to open or laparoscopic techniques. Standardization of reporting parameters, as advocated by Warren et al., has underscored the importance of reproducible techniques for meaningful outcome comparisons. In the specific context of flank hernia repair, laparoscopic series have shown acceptable outcomes but also technical limitations for large or laterally located defects [15, 16], while early robotic series demonstrated feasibility but lacked structured methods for tension management [1, 7]. Our technique builds upon the principle of intraoperative fascial traction, previously applied mainly in midline defects using external devices [8], and transfers it into a fully robotic, intracorporeal, stepwise closure strategy.

## Comparative and methodological considerations

Achieving tension-free fascial closure in large ventral or lateral defects often necessitates extensive component separation, most notably transversus abdominis release (TAR). While effective, TAR involves wide retromuscular dissection and longer operative times and carries a considerable risk of wound morbidity, seroma, hematoma, and chronic pain [14, 17]. Reported wound complication rates for component separation can reach up to 48% in soe series due to subcutaneous flap creation and muscle transection [18–20].

In contrast, PIFT preserves the integrity of the lateral abdominal wall by avoiding myofascial transection and extended dissection. The stepwise traction mechanism exploits the viscoelastic properties of the abdominal wall—creep and stress relaxation—to gradually reduce closure tension. This allows for approximation of fascial edges even in large defects, thereby circumventing the morbidity of formal release procedures.

The method is particularly attractive for lateral abdominal wall hernias, where defect orientation is typically transverse. By applying traction perpendicular to the muscle fiber direction, tissue compliance is utilized effectively. Adjunctive measures such as preoperative botulinum toxin A injection [20–24] or progressive pneumoperitoneum [25, 26] may further enhance closure feasibility in selected cases.

### Anatomical challenges at L1 and L3

Niebuhr et al. identified L1 and L3 defects as challenging for traction-based closure due to fixed bony insertions at the costal margins and iliac crest [8]. Several defects in our series were located in these anatomically constrained zones. Robotic access allows precise placement of widely spaced sliding-knot sutures, distributing traction forces evenly across the musculofascial layer. Sequential tightening at 10–15 min intervals promotes gradual tissue adaptation even where mobility is inherently limited. If required, peripheral adhesiolysis and limited retroperitoneal mobilization, including kidney mobilization, can provide additional length without formal component separation [13, 16, 27]. While closure of extreme L1/L3 defects may remain difficult, our experience indicates that PIFT extends the applicability of traction-based closure to lateral hernias previously considered unsuitable.

### Risk considerations

Closing the entire musculofascial layer under traction raises concerns regarding fascial tear, suture pull-through, and nerve entrapment. None of these complications occurred in our series. We attribute this to four factors:

- Use of multiple, widely spaced interrupted sutures with braided absorbable material to evenly distribute forces.
- Stepwise sliding-knot tightening, allowing progressive accommodation rather than abrupt loading.
- In line with current evidence and the European Hernia Society (EHS) guidelines, slowly absorbable monofilament sutures (such as PDS) are recommended for fascial closure [28]. These sutures provide similar tensile strength and incisional hernia outcomes as permanent (non-absorbable) sutures [29], without leaving permanent foreign material in situ. Notably, a meta-analysis of randomized trials found that using a slow-absorbing suture does not increase the risk of incisional hernia recurrence compared to non-absorbable sutures [30]. On the contrary, absorbable sutures can reduce long-term complications: for example, a Cochrane review reported that absorbable material significantly lowers the incidence of chronic wound sinus (persistent drainage) compared to permanent sutures [31]. Additionally, guidelines note that slowly absorbable sutures may decrease the risk of prolonged wound pain relative to permanent sutures [29]. A randomized trial found higher chronic pain rates with permanent Prolene than with absorbable Vicryl sutures (37% vs. 26%) [32].
- Mesh reinforcement after closure, ensuring long-term stability after suture resorption.

Nonetheless, our follow-up of six months limits conclusions on chronic pain and long-term nerve entrapment. These aspects extended follow-up in future studies.

### Clinical implications

The clinical outcomes observed are encouraging: low postoperative pain (median VAS 2), minimal complications (1 seroma, Clavien–Dindo I), no early recurrences, and short hospital stay (median 2 days). These results are consistent with recent multicenter data on robotic retromuscular repair reporting durable closure and low recurrence rates beyond one year [33]. Importantly, the technique is transferable to ventral hernias with transverse widths >7 cm, where it may offer a less invasive alternative to TAR, reducing operative morbidity while preserving mechanical integrity.

Functionally, preservation of the lateral abdominal wall may contribute to maintaining core stability and preventing long-term complications such as bulging or trunk imbalance, which are increasingly recognized in abdominal wall reconstruction [34–36]. The emphasis on function-preserving, minimally disruptive techniques aligns with current trends in robotic hernia surgery.

### Limitations

This study has inherent limitations. It is a single-center, retrospective series with a limited number of patients. The 6-month follow-up is sufficient for assessing early outcomes but does not allow for long-term conclusions on chronic pain, recurrence, or functional results. Prospective, multicenter studies with longer follow-up and comparative cohorts (e.g., TAR, IPOM) are needed to validate these findings.

## Conclusion

Robotic-assisted fascial closure using progressive sliding-knot traction with barbed-suture reinforcement represents a promising, physiologically sound, and minimally invasive approach to large flank and lateral hernias. By leveraging the viscoelastic behavior of the abdominal wall, this technique enables tension-reduced primary closure without formal component separation, while maintaining wall integrity. Early results demonstrate low morbidity, fast recovery, and no early recurrences. Further prospective studies are warranted to assess its long-term durability, functional outcomes, and comparative effectiveness.

## Supplementary Information

Below is the link to the electronic supplementary material.

Supplementary Material 1
